# Convergent biological pathways underlying the Kallmann syndrome-linked genes *Hs6st1* and *Fgfr1*

**DOI:** 10.1093/hmg/ddac172

**Published:** 2022-07-28

**Authors:** Sohyun Moon, Ying-Tao Zhao

**Affiliations:** Department of Biomedical Sciences, New York Institute of Technology College of Osteopathic Medicine, Old Westbury, NY 11568, USA; Department of Biomedical Sciences, New York Institute of Technology College of Osteopathic Medicine, Old Westbury, NY 11568, USA

## Abstract

Kallmann syndrome (KS) is a congenital disorder characterized by idiopathic hypogonadotropic hypogonadism and olfactory dysfunction. KS is linked to variants in >34 genes, which are scattered across the human genome and show disparate biological functions. Although the genetic basis of KS is well studied, the mechanisms by which disruptions of these diverse genes cause the same outcome of KS are not fully understood. Here we show that disruptions of KS-linked genes affect the same biological processes, indicating convergent molecular mechanisms underlying KS. We carried out machine learning-based predictions and found that KS-linked mutations in heparan sulfate 6-*O*-sulfotransferase 1 (*HS6ST1*) are likely loss-of-function mutations. We next disrupted *Hs6st1* and another KS-linked gene, fibroblast growth factor receptor 1 (*Fgfr1*), in mouse neuronal cells and measured transcriptome changes using RNA sequencing. We found that disruptions of *Hs6st1* and *Fgfr1* altered genes in the same biological processes, including the upregulation of genes in extracellular pathways and the downregulation of genes in chromatin pathways. Moreover, we performed genomics and bioinformatics analyses and found that Hs6st1 and Fgfr1 regulate gene transcription likely via the transcription factor Sox9/Sox10 and the chromatin regulator Chd7, which are also associated with KS. Together, our results demonstrate how different KS-linked genes work coordinately in a convergent signaling pathway to regulate the same biological processes, thus providing new insights into KS.

## Introduction

Kallmann syndrome (KS) is a congenital disorder characterized by idiopathic hypogonadotropic hypogonadism and olfactory dysfunction (1,2). Individuals with KS show symptoms such as incomplete or absent puberty, infertility, low levels of sex steroids, low levels of gonadotropin and loss of smell. The idiopathic hypogonadotropic hypogonadism is caused by deficits in the production, secretion or action of the gonadotropin-releasing hormone (GnRH), which is mainly because of defects in the development or migration of the GnRH producing neurons (3–5). The olfactory dysfunction, either absence of smell (anosmia) or partial loss of smell (hyposmia), is likely because of defects in the development of the olfactory system, such as impaired axon guidance of the olfactory neurons (6,7). Although some KS individuals can be treated with hormone-replacement therapy (2), many of the KS-associated symptoms are not treatable, which is in part due to our limited understanding of the molecular mechanisms underlying KS.

KS has a strong genetic basis (1,2,8,9). The first KS-linked gene, anosmin 1 (*ANOS1*, also known as *KAL1*), was identified in 1991 (10,11). Since then, studies in KS patients have identified KS-linked genetic variants in > 34 genes (1,2,4,5,8,12). Although the association between KS and several of these genes, such as fibroblast growth factor 8 (*FGF8*) (13) and heparan sulfate 6-*O*-sulfotransferase 1 (*HS6ST1*) (14), has been validated using animal models, the exact mechanisms by which these genes contribute to KS pathogenesis are not fully understood. Particularly, given that the KS-linked genes show diverse biological functions, how disruptions of these different genes cause the same outcome of KS remains elusive.

Most KS-linked genes show low penetrance and cause variable clinical severity (1,2,4,5,8). These findings lead to a proposed model of oligogenicity (15), in which mutations in multiple genes interact together to manifest a more severe phenotype. Indeed, large cohort studies revealed that >20% of individuals with KS or idiopathic hypogonadotropic hypogonadism are oligogenic (4,15). The oligogenicity of KS indicates that proteins encoded by the KS-linked genes might be involved in a same signaling pathway. For instance, *HS6ST1* (14), *FGF8* (13), *FGF17* (16) and *FGF receptor 1* (*FGFR1*) (17) are genetically linked to KS. HS6ST1 catalyzes the 6-*O*-sulfation on heparan sulfate (HS), a glycosaminoglycan that presents at the cell surface and extracellular matrix (ECM) of all human cells. Notably, HS 6-*O*-sulfation directly interacts with FGF and FGFR and enhances the bindings between the two (18–20), suggesting that HS6ST1, FGF8/FGF17 and FGFR1 belong to a same molecular pathway. However, it remains unclear how other KS-linked proteins, such as the transcription factor SOX10 (21) and the chromatin regulator CHD7 (22), contribute to this signaling pathway.

In this study, we disrupted *Hs6st1* and *Fgfr1* in mouse neuronal cells and carried out RNA sequencing (RNA-seq) to assess the transcriptome changes upon the disruptions. We found that disruptions of *Hs6st1* and *Fgfr1* altered the expression of genes in the same biological processes, suggesting that the two genes belong to a same molecular pathway. We also found that Hs6st1 and Fgfr1 regulate gene transcription likely via Sox9 and Chd7. Taken together, our results indicate a convergent signaling pathway underlying multiple KS-linked genes, thus providing novel insights into KS.

## Results

### KS-linked mutations in HS6ST1 affect the protein structure

**Figure 1:**
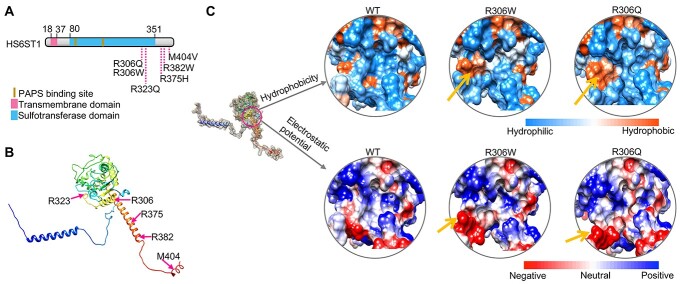
KS-linked mutations in HS6ST1 affect the protein structure. (**A**) Diagram of the human HS6ST1 protein and the six KS-linked mutations. (**B**) Three-dimensional protein structure of human HS6ST1 predicted using the AlphaFold. (**C**) The hydrophobicity and electrostatic potential of the WT and two mutant HS6ST1 proteins. Yellow arrows indicate the loci of property changes at the protein surface.

Mutations in HS6ST1 are genetically linked to KS (14) (Fig. 1A), but the underlying mechanisms are not fully understood. To determine how KS-linked mutations affect HS6ST1 function, we assessed the effects of these point mutations on the protein structure of HS6ST1 using a novel machine learning-based approach. We used the deep learning algorithm in the AlphaFold software (23) to predict the three-dimensional structures of the wild-type (WT) and mutant HS6ST1. We found that the WT HS6ST1 has 11 α-helices and four β-strands (Fig. 1B and Supplementary Material, Fig. S1), which is similar to the crystal structure of the zebrafish Hs6st3 catalytic domain (24). We also used the AlphaFold to predict the structures of the mutant HS6ST1 (Supplementary Material, Fig. S2). We found that the KS-linked mutations affect the local properties of the protein (Fig. 1C and Supplementary Material, Fig. S2), but they do not alter the global protein structures (Supplementary Material, Fig. S2). For example, R306W and R306Q, which localize at the ninth α-helix of the protein, increase the hydrophobicity and negative electrostatic potential of a valley-like structure at the catalytic core of the protein (Fig. 1C and Supplementary Material, Fig. S2). Thus, these two mutations likely affect the enzyme activity. This finding is consistent with previous *in vitro* biochemical studies that the R306Q, R306W, R382W, M404V and R375H mutations reduce the enzyme activities (14,24,25). In contrast, no obvious alternation of protein surface properties was observed in the R323Q HS6ST1 (Supplementary Material, Fig. S2). Notably, the R323 localizes in the substrate-binding pocket of the protein and interacts with the cofactor product 3′-phosphoadenosine 5′-phosphate (24). Thus, the R323Q mutation may directly abolish the substrate recognition or binding instead of altering the protein structure, which is also consistent with the *in vitro* biochemical studies (14,24). Together, these results suggest that KS-linked mutations in HS6ST1 affect the protein structures and are likely loss-of-function mutations.

### Disruption of *Hs6st1* in Neuro 2a cells alters the transcriptome

**Figure 2:**
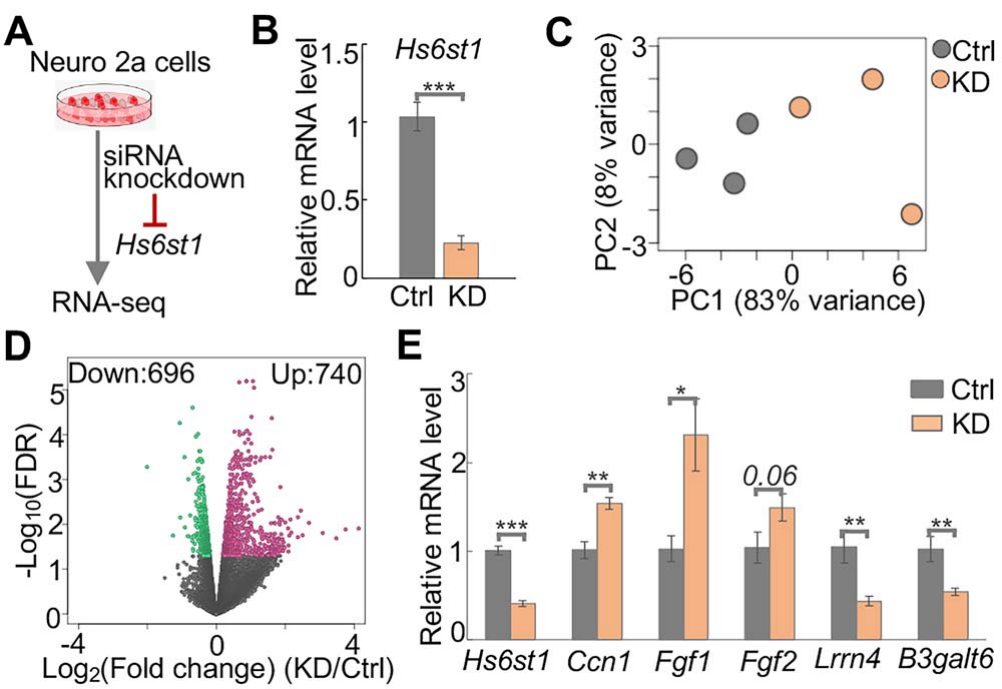
*Hs6st1* knockdown alters the transcriptome. (**A**) Diagram of the experiment design. (**B**) qPCR results showing the relative mRNA levels (2^-ΔΔCt^ values) of *Hs6st1* in the control (Ctrl) and *Hs6st1* knockdown (KD) samples. Ctrl, *n* = 3; KD, *n* = 3. ^*^^*^^*^P-value < 0.001, one-tailed *t*-test. (**C**) Principal component (PC) analysis results. (**D**) Volcano plot showing the fold change and FDR. Red dots represent the 740 upregulated genes, and green dots represent the 696 downregulated genes. (**E**) qPCR results showing the relative mRNA levels (2^-ΔΔCt^ values) of six genes in the independent experiments of *Hs6st1* knockdown. Ctrl, *n* = 2; KD, *n* = 8. ^*^P-value < 0.05; ^*^^*^P-value < 0.01; ^*^^*^^*^P-value < 0.001; one-tailed *t*-test.

To illustrate the molecular mechanisms by which HS6ST1 dysfunction contributes to KS, we disrupted *Hs6st1* in mouse neuronal cells and assessed the transcriptome changes (Fig. 2A). We used Neuro 2a cells, a widely used mouse neuroblastoma cell line that expresses high levels of *Hs6st1* and low levels of the other *Hs6st* genes (Supplementary Material, Fig. S3A). To disrupt *Hs6st1*, we transfected Neuro 2a cells with siRNAs that specifically target the messenger RNA (mRNA) of *Hs6st1*. Neuro 2a cells transfected with control siRNAs against a scrambled sequence were used as the control. We found that siRNA treatment decreased the mRNA levels of *Hs6st1* by 78% (Fig. 2B, *P-*value < 0.001, one-tailed *t*-test).

To assess the transcriptome changes upon the knockdown of *Hs6st1*, we performed ribosomal RNA-depleted RNA sequencing (RNA-seq) experiment. We sequenced three biological replicates for the control and *Hs6st1* knockdown samples and obtained 517 million high-quality sequencing reads for the six RNA-seq libraries (Supplementary Material, Fig. S3B). We next used a computational pipeline to analyze the RNA-seq data and quantified the expression levels of all genes in the mouse mm10 genome (described in detail in the Materials and Methods section). We found a high correlation among the biological replicates (Pearson’s correlation coefficient r > 0.994). To further assess the six transcriptome profiles, we performed a principal component analysis (PCA). We found a clear separation of the three control samples and the three *Hs6st1* knockdown samples (Fig. 2C), suggesting that the siRNA knockdown of *Hs6st1* consistently alters the transcriptome.

To identify genes that were affected by the *Hs6st1* knockdown, we compared the expression profiles of all genes between the control and the knockdown RNA-seq data using edgeR (26). We identified 1436 genes that were significantly affected by the *Hs6st1* knockdown (false discovery rate (FDR) < 0.05) (Fig. 2D, Supplementary Material, Fig. S3C and Supplementary Material, Table S1), including 740 upregulated genes and 696 downregulated genes (Fig. 2D). For example, the cellular communication network factor 1 (*Ccn1*) and two *Fgf* genes (*Fgf1* and *Fgf2*) were upregulated by the knockdown, whereas the leucine rich repeat neuronal 4 (*Lrrn4*) and the beta-1,3-galactosyltransferase 6 (*B3galt6*) were downregulated (Supplementary Material, Fig. S3D).

To validate the gene expression changes upon *Hs6st1* knockdown, we carried out independent experiments to knock down *Hs6st1* using the same siRNAs and measured the expression changes of six genes using quantitative reverse transcription PCR (qPCR). We found a significant decrease of the mRNA levels of *Hs6st1* (Fig. 2E), supporting the knockdown efficiencies. For the other five genes, the expression levels of *Ccn1*, *Fgf1* and *Fgf2* were increased, and the expression levels of *Lrrn4* and *B3galt6* were decreased in the knockdown experiments (Fig. 2E). These results are consistent with the results from the RNA-seq (Supplementary Material, Fig. S3D). Notably, we also found the increased expression of *Ccn1* and the decreased expression of *Lrrn4* in the brain of the *Hs6st1* nervous system-specific knockout mice (data not shown). Together, these results suggest that knockdown of *Hs6st1* in Neuro 2a cells alters the transcriptome.

### *Hs6st1* knockdown promotes genes in the extracellular pathways and inhibits genes in the chromatin pathways

**Figure 3:**
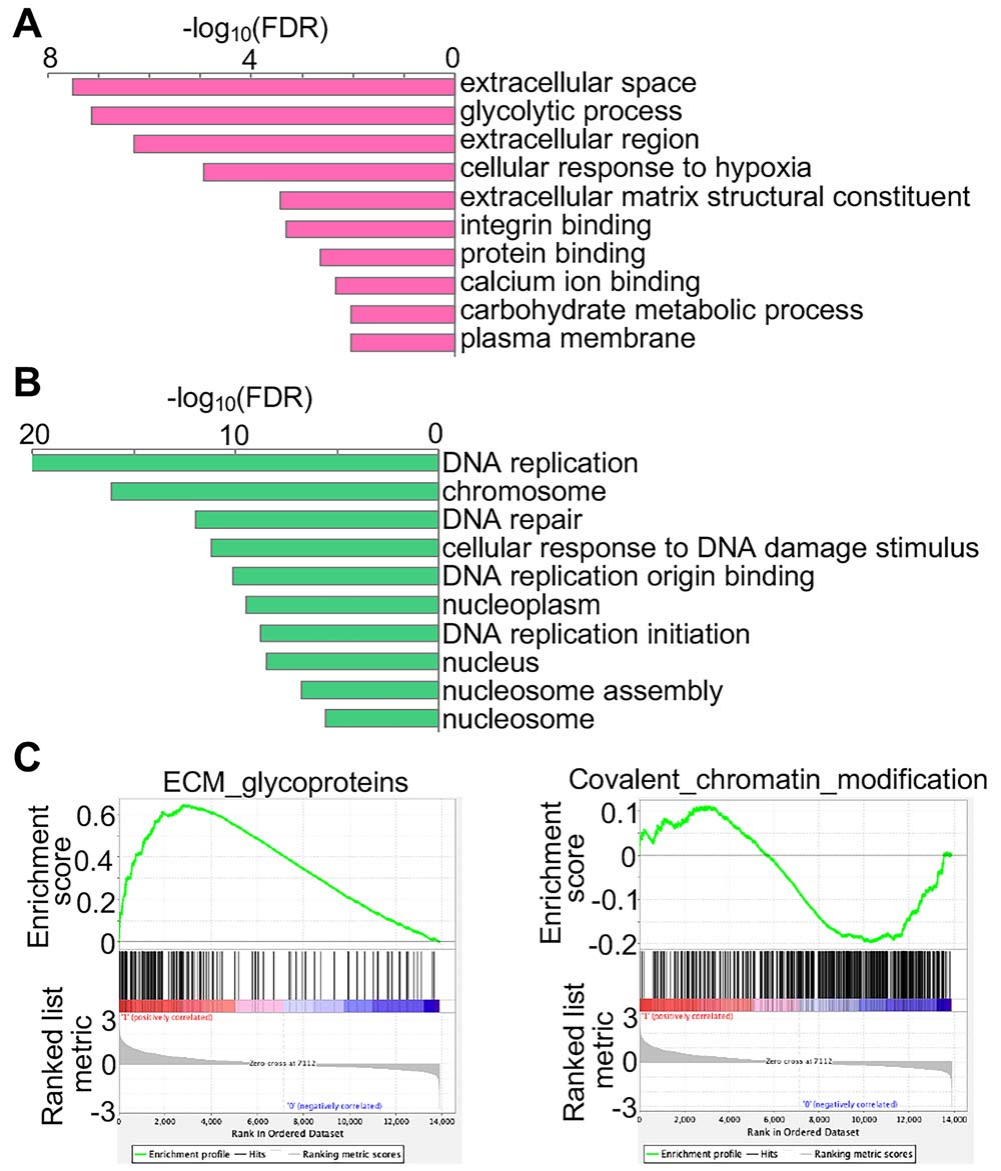
*Hs6st1* knockdown promotes genes in the extracellular pathways and inhibits genes in the chromatin pathways. (**A**) Gene Ontology pathways enriched by the upregulated genes upon *Hs6st1* knockdown. (**B**) Gene Ontology pathways enriched by the downregulated genes upon *Hs6st1* knockdown. (**C**) GSEA results showing the upregulation of the ECM glycoproteins pathway and the downregulation of the covalent chromatin modification pathway.

To determine the biological pathways affected by the *Hs6st1* knockdown, we carried out Gene Ontology enrichment analysis (27) and Gene Set Enrichment Analysis (GSEA) (28). We found that the upregulated and downregulated genes were enriched for distinct gene ontology pathways (FDR < 0.05). The 740 upregulated genes were enriched for extracellular pathways, such as glycolytic process, ECM structural constituent and integrin binding (Fig. 3A). In contrast, the 696 downregulated genes were enriched for chromatin pathways, such as DNA replication, chromosome, DNA repair and nucleosome assembly (Fig. 3B). The GSEA results also showed that extracellular pathways, such as the ECM glycoproteins, were upregulated, whereas chromatin pathways, such as the covalent chromatin modification, were downregulated (Fig. 3C). Together, these results indicate that *Hs6st1* knockdown promotes genes in the extracellular pathways and inhibits genes in the chromatin pathways.

### Disruption of *Fgfr1* in Neuro 2a cells alters the transcriptome

**Figure 4:**
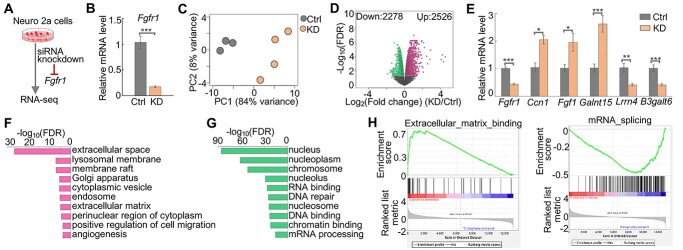
*Fgfr1* knockdown alters the transcriptome. (**A**) Diagram of the experiment design. (**B**) qPCR results showing the relative mRNA levels (2^-ΔΔCt^ values) of *Fgfr1* in control (Ctrl) and *Fgfr1* knockdown (KD) samples. Ctrl, *n* = 3; KD, *n* = 5. ^*^^*^^*^P-value < 0.001, one-tailed *t*-test. (**C**) Principal component (PC) analysis results. (**D**) Volcano plot showing the fold change and FDR. Red dots represent the 2526 upregulated genes, and green dots represent the 2278 downregulated genes. (**E**) qPCR results showing the relative mRNA levels (2^−ΔΔCt^ values) of six genes in the independent experiments of *Fgfr1* knockdown. Ctrl, *n* = 2; KD, *n* = 7. ^*^P-value < 0.05; ^*^^*^P-value < 0.01; ^*^^*^^*^P-value < 0.001; one-tailed *t*-test. (**F**) Gene Ontology pathways enriched by the upregulated genes upon *Fgfr1* knockdown. (**G**) Gene Ontology pathways enriched by the downregulated genes upon *Fgfr1* knockdown. (**H**) GSEA results showing the upregulation of the ECM binding pathway and the downregulation of the mRNA splicing pathway.

To determine the extent to which the biological pathways affected by the *Hs6st1* disruption are shared among other KS-linked genes, we next carried out siRNA knockdown and RNA-seq experiments for another KS-linked gene, *Fgfr1* (Fig. 4A). Neuro 2a cells express high levels of *Fgfr1* and low levels of the other *Fgfr* genes (Supplementary Material, Fig. S3E). To disrupt *Fgfr1*, we transfected Neuro 2a cells with siRNAs that specifically target *Fgfr1* mRNA. We found that siRNA treatment decreased the mRNA levels of *Fgfr1* by 83% (Fig. 4B, *P-*value < 0.001, one-tailed *t*-test). We then carried out RNA-seq experiment with four biological replicates (Supplementary Material, Fig. S3B). The PCA showed a clear separation of the three control samples and the four *Fgfr1* knockdown samples (Fig. 4C), suggesting that siRNA knockdown of *Fgfr1* consistently alters the transcriptome.

To identify genes that were affected by the *Fgfr1* knockdown, we used our RNA-seq analysis pipeline and identified 2526 upregulated genes and 2278 downregulated genes (FDR < 0.05) (Fig. 4D, Supplementary Material, Fig. S3F and Supplementary Material, Table S2). To validate the gene expression changes upon *Fgfr1* knockdown, we carried out independent experiments to knock down *Fgfr1* using the same siRNAs and measured the expression changes of *Fgfr1* and five other genes using qPCR. We found a significant decrease of the mRNA levels of *Fgfr1* (Fig. 4E), supporting the knockdown efficiencies. The qPCR results of the other five genes are consistent with the RNA-seq results, including the upregulation of the *Ccn1*, *Fgf1* and polypeptide *N*-acetylgalactosaminyltransferase 15 (*Galnt15*) and the downregulation of the *Lrrn4* and *B3galt6* (Fig. 4E and Supplementary Material, Fig. S3G). Together, these results suggest that knockdown of *Fgfr1* in Neuro 2a cells alters the transcriptome.

To determine the biological pathways affected by the *Fgfr1* knockdown, we carried out Gene Ontology enrichment analysis and GSEA analysis. We found that the upregulated genes were enriched for extracellular pathways (Fig. 4F), and the downregulated genes were enriched for chromatin pathways (Fig. 4G). The GSEA results also showed that extracellular pathways, such as the ECM binding, were upregulated, whereas chromatin pathways, such as the mRNA splicing, were downregulated (Fig. 4H). Notably, the upregulated pathways and downregulated pathways are in the same biological processes between the *Hs6st1* knockdown (Fig. 3A–C) and the *Fgfr1* knockdown (Fig. 4F–H). Together, these results indicate that *Hs6st1* knockdown and *Fgfr1* knockdown affect the same biological processes.

### A group of 1052 genes are regulated by both Hs6st1 and Fgfr1

**Figure 5:**
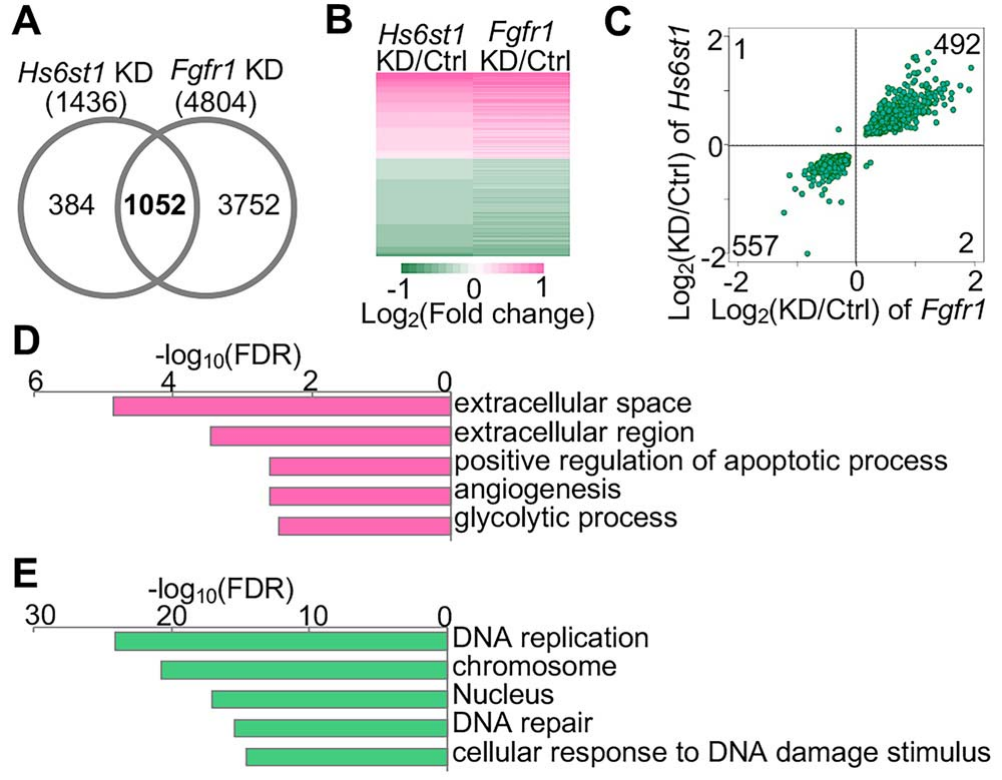
A group of 1052 genes are regulated by both Hs6st1 and Fgfr1. (**A**) Comparison of the differentially expressed genes (DEGs) between the *Hs6st1* knockdown (KD) and *Fgfr1* KD. Note, 1052 DEGs were overlapped. (**B**) Heatmap showing the expression changes of the 1052 overlapping DEGs in the two KD experiments. (**C**) Scatter plot showing the fold changes of the 1052 overlapping DEGs in the two KD experiments. (**D**) Gene Ontology pathways enriched by the 492 upregulated overlapping DEGs. (**E**) Gene Ontology pathways enriched by the 557 downregulated overlapping DEGs.

To identify genes that are regulated by both Hs6st1 and Fgfr1, we compared the two lists of differentially expressed genes (DEGs) in the two knockdown experiments. We found that 1052 genes were affected in both knockdown experiments (Fig. 5A and Supplementary Material, Table S3), which we term overlapping DEGs. To determine the expression changes of the 1052 overlapping DEGs in the two knockdown experiments, we analyzed their values of fold changes. We found that 99.7% (1049 out of 1052) of the 1052 overlapping DEGs showed the same directions of the gene expression changes upon *Hs6st1* knockdown and *Fgfr1* knockdown (Fig. 5B and C). These results suggest that Hs6st1 and Fgfr1 belong to a same signaling pathway to regulate the 1052 genes. To determine the biological pathways of the 1052 overlapping DEGs, we carried out Gene Ontology enrichment analysis. We found that the 492 upregulated genes were enriched for extracellular pathways (Fig. 5D), whereas the 557 downregulated genes were enriched for chromatin pathways (Fig. 5E). Together, these results suggest that a group of 1052 genes are regulated by both Hs6st1 and Fgfr1.

### Hs6st1 and Fgfr1 regulate gene transcription likely via the transcription factor Sox9

**Figure 6:**
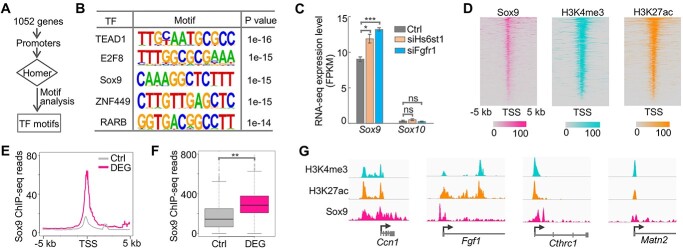
Hs6st1 and Fgfr1 regulate gene transcription likely via the transcription factor Sox9. (**A**) The pipeline of motif analysis. (**B**) The top five identified motifs. (**C**) Expression profiles of *Sox9* and *Sox10* upon *Hs6st1* knockdown (siHs6st1) and *Fgfr1* knockdown (siFgfr1). ^*^P-value < 0.05; ^*^^*^^*^P-value < 0.001; ns, *P-*value > 0.05; one-tailed *t*-test. (**D**) Heatmaps of ChIP-seq profiles of Sox9, H3K4me3 and H3K27ac at the promoter regions of the 1052 overlapping DEGs. TSS, transcription start site. (**E**) Sox9 ChIP-seq profiles at the promoter regions of the 1052 overlapping DEGs and the 1000 control genes (Ctrl). (**F**) Boxplot of Sox9 ChIP-seq sequencing reads at the promoter regions. ^*^^*^P-value = 0.001; one-tailed *t*-test. (**G**) Snapshots of ChIP-seq profiles at four DEGs.

Although Hs6st1 and Fgfr1 regulate the transcription of the 1052 genes (Fig. 5), the transcription factors involved in this process remain unknown. To identify transcription factors that are downstream of Hs6st1 and Fgfr1, we first carried out a Homer motif analysis (29) using the promoter regions of the 1052 overlapping DEGs (Fig. 6A). We found that these promoter regions were enriched for 16 *de novo* motifs (Supplementary Material, Fig. S3H). Figure 6B shows the top five *de novo* motifs identified using Homer, including the transcription factor Sox9. Sox9 belongs to the Sox-E transcription factor family, which also includes Sox10 (30). Notably, mutations in *SOX10* are also genetically linked to KS (21). Furthermore, we found that although *Sox10* is lowly expressed in Neuro 2a cells, *Sox9* was significantly upregulated upon the *Hs6st1* knockdown and the *Fgfr1* knockdown (Fig. 6C).

To assess the genomic bindings of Sox9, we analyzed the publicly available genome-wide binding profiles of Sox9 (31), which were generated by chromatin immunoprecipitation followed by sequencing (ChIP-seq). We first analyzed Sox9 ChIP-seq signals at the promoter regions of the 1052 overlapping DEGs and found an enrichment of ChIP-seq signals (Fig. 6D), suggesting that Sox9 directly binds to the promoters of these genes. In addition, to assess the chromatin environment of these promoters, we analyzed our previously generated ChIP-seq profiles of histone H3 trimethylation at lysine 4 (H3K4me3) and histone H3 acetylation at lysine 27 (H3K27ac) (32). We found an enrichment of H3K4me3 and H3K27ac ChIP-seq signals at the promoter regions of the 1052 overlapping DEGs (Fig. 6D), suggesting that these promoters are in active chromatin status. Furthermore, to determine whether the Sox9 bindings are specific to the 1052 overlapping DEGs, we randomly selected 1000 control genes that were expressed in Neuro 2a cells but were not affected by the two knockdown experiments. We found a significant enrichment of Sox9 ChIP-seq signals at the promoters of the 1052 overlapping DEGs compared with the 1000 control genes (Fig. 6E and F  *P-*value = 0.001, one-tailed *t*-test), indicating that Sox9 bindings are specific to the DEGs. We also showed the ChIP-seq profiles at four DEGs as an example (Fig. 6G). Lastly, although *Sox10* was not affected by the two knockdown experiments (Fig. 6C), we analyzed the publicly available Sox10 ChIP-seq data (33) and found an enrichment of Sox10 bindings at the promoters of the 1052 overlapping DEGs (Supplementary Material, Fig. S3I). Together, these results indicate that Sox9 is likely the transcription factor downstream of Hs6st1 and Fgfr1.

### A convergent signaling pathway underlying the KS-linked genes

**Figure 7:**
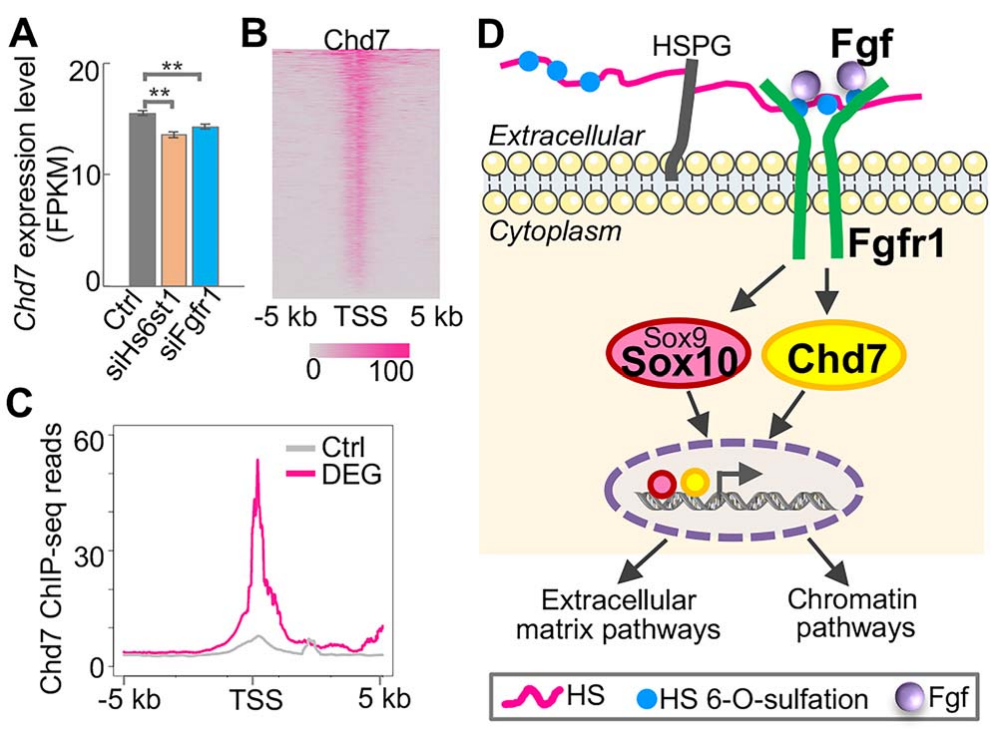
A convergent signaling pathway underlying the KS-linked genes. (**A**) Expression profiles of the chromatin regulator Chd7 upon the two knockdown experiments. ^*^^*^P-value < 0.01; one-tailed *t*-test. (**B**) Heatmap of Chd7 ChIP-seq profiles at the promoter regions of the 1052 overlapping DEGs. (**C**) Chd7 ChIP-seq profiles at the promoter regions of the 1052 overlapping DEGs and the 1000 control genes (Ctrl). (**D**) Proposed model of the convergent pathway underlying the KS-linked genes *Hs6st1*, *Fgf*, *Fgfr1*, *Sox10* and *Chd7*. HS, heparan sulfate. HSPG, heparan sulfate proteoglycan.

Thirty-four genes are genetically linked to KS (1,2,4,5,8,12) (Supplementary Material, Fig. S3J). Given that the three KS-linked genes, *Hs6st1*, *Fgfr1*, and likely *Sox10*, regulate the same biological processes, we next asked whether other KS-linked genes also play a role in these pathways. To answer this question, we first analyzed the expression changes of the 34 KS-linked genes upon *Hs6st1* knockdown and *Fgfr1* knockdown. We found that except *Hs6st1* and *Fgfr1*, three other KS-linked genes, chromodomain helicase DNA binding protein 7 (*Chd7*), dual specificity phosphatase 6 (*Dusp6*) and coiled-coil domain containing 141 (*Ccdc141*), were also significantly affected by the knockdown experiments (Supplementary Material, Tables S1 and S2). For instance, *Chd7*, which encodes a chromatin regulator, was significantly downregulated (Fig. 7A). Given that disruptions of *Hs6st1* and *Fgfr1* inhibit chromatin processes (Fig. 5E), we next examined whether these inhibitions are mediated by Chd7. Thus, to determine the binding profiles of Chd7, we analyzed the publicly available Chd7 ChIP-seq data (34). We found an enrichment of Chd7 bindings at the promoters of the 1052 overlapping DEGs (Fig. 7B and C), suggesting that Chd7 may modify the chromatin status of these genes to regulate their expression. In addition, we found that genes affected by the knockdown of *Hs6st1* and *Fgfr1* tend to exhibit the same directions of expression changes in the brain of *Chd7* knockout mice (34) (Supplementary Material, Fig. S3K).

Together, our results indicate a model by which multiple KS-linked genes, including *Hs6st1*, *Fgf*, *Fgfr1*, *Sox10* and *Chd7*, converge on a same signaling pathway (Fig. 7D). In this model, Hs6st1 catalyzes the HS 6-*O*-sulfation to control the HS/FGF/FGFR interactions to regulate the expression of *Sox9* and *Chd7*. Sox9 upregulation will promote the ECM pathways, which has been reported by a previous study (35). Chd7 downregulation will modify chromatin status to inhibit the chromatin pathways.

## Discussion

KS, once considered as a monogenic disorder, has been linked to >34 genes (1,2,4,5,8,12). In this study, we found that multiple KS-linked genes, including *HS6ST1*, *FGF*, *FGFR1*, *SOX10* and *CHD7*, work coordinately in the same signaling pathway. To determine KS-linked mutations in HS6ST1, we carried out machine learning-based predictions and found that KS-linked mutations in *HS6ST1* are likely loss-of-function mutations. In addition, by carrying out siRNA knockdown and RNA-seq, we found that disruptions of *Hs6st1* and *Fgfr1* in mouse neuronal cells affected the same biological processes. Lastly, by performing genomics and bioinformatics analyses, we found that Hs6st1 and Fgfr1 regulate gene transcription likely via the transcription factor Sox9/Sox10 and the chromatin regulator Chd7. Taken together, our study indicates a convergent signaling pathway underlying multiple KS-linked genes.

ECM pathways in the brain play an important role in the regulation of brain development and function, such as neuron migration, axon guidance and synapse maturation (36). Intriguingly, defects in the migration and development of the GnRH neurons and the olfactory neurons are the two hallmarks of KS (3–7). In addition, the first identified KS gene, *ANOS1*, encodes a glycoprotein that plays an important role in ECM pathways (10,11). In this study, we found that disruptions of *Hs6st1* and *Fgfr1* in mouse neuronal cells both promote the expression of genes in the ECM pathways. Thus, our findings highlight a role of ECM pathways in the KS pathogenesis. Moreover, we found that disruptions of *Hs6st1* and *Fgfr1* upregulated the expression of the transcription factor Sox9. Given that Sox9 directly promotes the expression of ECM genes in the brain (35), our findings together suggest a model by which the HS/FGFR signaling regulates Sox9 expression to promote the ECM processes. Further studies are needed to directly dissect the role of Sox9 and the ECM pathways in the pathogenesis of KS.

SOX9 belongs to the E subgroup of the SOX transcription factor family (SOX-E). SOX-E family includes three members, SOX8, SOX9 and SOX10, which exhibit similar DNA binding motifs and function redundancy (30,37,38). SOX-E transcription factors play an important role in development. Notably, mutations in *SOX10* have been found in KS individuals (21). In this study, we found a central role of Sox9 in the signaling pathways that are critical to KS. Therefore, it will be intriguing to investigate genetic variants in *SOX9* as well as *SOX8* in KS individuals.

We identified that disruptions of *Hs6st1* and *Fgfr1* in mouse neuronal cells both downregulated genes in chromatin pathways, such as chromatin modification, nucleosome assembly, DNA binding and chromatin binding. Given that Hs6st1 and Fgfr1 are localized at the Golgi and the cell surface, they are unable to directly regulate biological processes in the nucleus. One possible mediator is Chd7, which is a chromatin remodeling enzyme that plays an important role in promoting chromatin accessibility, enhancer activation and active histone modifications in the brain (34,39). We found that disruptions of *Hs6st1* and *Fgfr1* downregulated the expression of *Chd7*, suggesting a model by which the HS/FGFR signaling regulates *Chd7* expression to inhibit the chromatin processes. Notably, a previous study found that Chd7 interacts and cooperates with Sox10 to regulate chromatin status and gene transcription in the brain (39). Thus, further investigations are needed to reveal how the two KS-linked proteins, Chd7 and Sox10, and the chromatin pathways contribute to KS pathogenesis.

Oligogenicity is a key feature of the genetic basis of KS. Genetic interactions among KS-linked genes have been reported by previous studies. Many of these interactions are centering on the FGFR signaling. For instance, ANOS1 functionally interacts with the FGFR–FGF–HS complex in human embryonic GnRH olfactory neuroblasts (40). ANOS1 function in *Caenorhabditis elegans* also requires FGFR and HS 6-*O*-sulfation (14). In addition, the localization of interleukin 17 receptor D (IL17RD), a KS-linked protein, in the olfactory placode requires FGF8 and the FGFR signaling (16). Furthermore, *Dusp6* is linked to KS and is a negative feedback regulator of the FGF/FGFR signaling in mouse development (41). The β-Klotho (KLB) function also needs the FGF/FGFR signaling (42). Notably, some KS-linked genes can interact independent of the FGFR signaling. For instance, Chd7 and Sox10 work corporately to regulate the myelination in the mouse brain. In this study, we found that multiple KS-linked genes, including Hs6st1, Fgf, Fgfr1, Chd7, Sox10 and likely Dusp6, converge on a same signaling pathway to regulate genes in the ECM processes and chromatin processes. Together, these findings indicate a potential gene interaction network among KS-linked genes. Further studies are needed to determine whether and how other KS-linked genes involve in this network and contribute to the oligogenicity.

In summary, our data demonstrate convergent molecular pathways underlying the KS-linked genes. Moving forward, we propose that these convergent pathways may involve other KS-linked genes and may directly contribute to the etiology of KS. Given that this study is focused on the signaling pathways underlying KS using neuronal cell lines, the role of these signaling pathways in KS pathogenesis, such as deficits in neuron migration and axon guidance, remains elusive. As such, we think further investigations are warranted to reveal the association and the detailed molecular mechanisms of these convergent pathways in KS pathogenesis in animal models and in KS individuals. Furthermore, to validate the transcriptome changes identified in this study, future investigations are needed to assess and integrate the transcriptome of GnRH neurons and olfactory neurons from KS patients and KS animal models, which are still lacking at present. Given that the six KS-linked variants in *HS6ST1* may affect different aspects of the HS6ST1 protein function, future investigations, such as variant-specific rescue experiments in *Hs6st1* disrupted neurons, are needed to pinpoint the common and variant-specific effects. Lastly, these convergent pathways also could be a potential therapeutic target for KS.

## Materials and Methods

### Cell cultures

Neuro 2a cells were purchased from the ATCC (CCL-131). The cells were cultured and maintained in Dulbecco’s modified Eagle’s medium (DMEM, Gibco # 11965092) and were supplemented with 10% fetal bovine serum (FBS, Gibco # 26140079), 2 mM L-glutamine (Gibco # 25030081), 100 U/ml penicillin and 100 mg/ml streptomycin (Gibco # 15240062). The cells were incubated at 37°C in a humidified atmosphere incubator containing 5% CO_2_. The cells were sub-cultured in fresh medium after reaching 70–80% confluence (about every 2–3 days).

### siRNA knockdown

The siRNA transfection of Neuro 2a cells was performed using siRNA transfection reagent (Santacruz Biotech, sc-29 528) according to the manufacturer’s instructions. The siRNAs used in this study include siRNAs for *Hs6st1* (Santacruz Biotech, sc-146 089), siRNAs for *Fgfr1* (Santacruz Biotech, sc-29 317) and control siRNA (Santacruz Biotech, sc-37 007). Briefly, for each transfection, Neuro 2a cells were plated for 1 day and then were incubated with siRNA (60 pmol), the transfection reagent and the transfection medium for 6 h at 37°C incubator. After the incubation, the cells were overlaid with the same volume of fresh growth medium containing 10% FBS and were cultured overnight in 37°C incubator. In the next day, the medium was exchanged to fresh growth medium containing 10% FBS, and the cells were incubated at 37°C, 5% CO_2_ for an additional 72 h before harvesting for analysis.

### RNA isolation and quality assessment

RNA was isolated from Neuro 2a cells, and the total RNA was then extracted using the Trizol reagent (Invitrogen, #15596026) following the manufacturer’s instruction. After the isolation, the quality, concentration and integrity of the isolated RNA were assessed using the Nanodrop spectrometer and the Bioanalyzer (Agilent 2100) using RNA 6000 Nano Chip. RNA samples with a RIN value of > 8 were included for the downstream experiments.

### RNA-seq library preparation, quality control and sequencing

RNA-seq libraries were prepared according to the manufacturer instructions with the Stranded Total RNA Library Prep kit (Zymo, #R3003). The libraries were prepared with 1000 ng of total RNAs. Library quality was confirmed by the Bioanalyzer (Agilent 2100) using DNA 7500 chip. The fragment sizes of all libraries were within the range of 300–500 bp. The libraries were sequenced using the Illumina NovaSeq sequencer.

### qPCR

To assess the relative mRNA levels, after RNA isolation, cDNA was synthesized with Prime script reverse transcription kit (Takara, #RR037A). Real-time PCR was performed using the SYBR Green PCR Master Mix (Applied Biosystems, #4309155). The 18S rRNA was used as the endogenous reference. The primers used were listed as follows:*B3galt5* forward, 5′-GGCAACTCTGCGACTACTAC-3′; *B3galt5* reverse, 5′-CGATACGTCTTCACTGTGC-3′; *Ccn1* forward, 5′-ATGAAGACAGCATTAAGGACTC-3′; *Ccn1* reverse, 5′-TGCAG AGGGTTGAAAAGAAC-3′; *Fgf1* forward, 5′-CCTGCCAGTTCTTCAG TGC-3′; *Fgf1* reverse, 5′-GGCTGCGAAGGTTGTGAT-3′; *Fgf2* forward, 5′-CACCAGGCCACTTCAAGGA-3′; *Fgf2* reverse, 5′-GATGGATGCGC AGGAAGAA-3′; *Fgfr1* forward, 5′-GCCTCACATTCAGTGGCTGAAG-3′; *Fgfr1* reverse, 5’-AGCACCTCCATTTCCTTGTCGG-3′; *Galnt15* forward, 5′-TGGCCAATGTCTACCCTGAG-3′; *Galnt15* reverse, 5′-CACA GAGGCCAAATCCAGTG-3′; *Hs6st1* forward, 5′-TGGACCGAACTCAC CAACTGTG-3′; *Hs6st1* reverse, 5′-CATTCACTCAGGTAGCGGGATAC-3′; *Lrrn4* forward, 5′-TGAGTTCCTTTGGTCCTTGG-3′; *Lrrn4* reverse, 5′-TGTTGACATCCACGAGAAGC-3′; *Hs6st2* forward, 5′-ACTCTCCA TCCTCCACAAAGCC-3′; *Hs6st2* reverse, 5′-CCAAGTTGCTCCTCTCT GGACA-3′; *Hs6st3* forward, 5′-CTGGACCGAGCTCACCAAC-3′; *Hs6st3* reverse, 5′-CGTCGCACATATGGAGAGAGGT-3′; 18S rRNA forward, 5′-GTAACCCGTTGAACCCCATT-3′; 18S rRNA reverse, 5′-CCATCCAATCGGTAGTAGCG-3′. The cycling parameters used for qPCR amplification reactions were: AmpliTaq activation at 95°C for 10 min, denaturation at 95°C for 15 s and annealing/extension at 60°C for 1 min (40 cycles).

### qPCR quantification and statistical analyses

The cycle threshold (Ct) values were obtained from the StepOnePlus real-time PCR system (Applied Biosystems) for both the endogenous reference gene (18S rRNA) and target genes. The Microsoft Excel was used for the downstream data analyses. For each sample, we had two to three technical replicates and two to eight biological replicates. First, we calculated the mean Ct value for the 18S rRNA for each sample ($\bar{\textrm{x}} $Ct_18S_) on the basis of the 18S rRNA Ct values of different technical replicates of that sample. We next calculated the ΔCt values for target genes in the control and knockdown samples by subtracting $\bar{\textrm{x}} $Ct_18S_ of each sample from the Ct values of these target genes. We then calculated the mean ΔCt value of control sample ($\bar{\textrm{x}} $ΔCt_control_) using the ΔCt values of the control samples of all biological and technical replicates. To calculate the ΔΔCt values, we subtracted $\bar{\textrm{x}} $ΔCt_control_ from the ΔCt values of target genes in the control and knockdown samples. Lastly, we transformed the ΔΔCt values into 2^−ΔΔCt^ values. We used the 2^−ΔΔCt^ values to calculate the mean and standard error and to plot. We used the ΔCt values and the Excel t.test (one-tailed, two-sample unequal variance) to calculate the *P*-values for the gene expression differences between control and knockdown samples.

### Protein structure prediction and visualization

The AlphaFold v2.0 (23) was used to predict the protein structures. The human HS6ST1 protein sequence was obtained from the National Center for Biotechnology Information (NCBI) database by the accession number of NP_004798.3. For mutant proteins, the according amino acid was replaced by the mutant allele. The AlphaFold docker was installed. The AlphaFold was carried out using the default parameters except for the ‘—max_template_date = 2020-05-14’. The ‘ranked_0.pdb’ file was selected as the predicted protein structure. The UCSF Chimera (43) was used to visualize the protein structures.

### RNA-seq alignment and read counting

The RNA-seq data analyses were performed as previously described (44–48). Briefly, the FASTQ files of RNA-seq were aligned to the mouse mm10 genome using STAR 2.7.7a (49) by the parameters of ‘--runThreadN 40 --outFilterMultimapNmax 1 --outFilterMismatchNmax 3 --outFilterScoreMinOverLread 0.25 --outFilterMatchNminOverLread 0.25’. To increase the speed of the alignment, we used the ‘—runThreadN 40’ parameter to use 40 processors/CPUs. To exclude ambiguously mapped reads, we used the ‘—outFilterMultimapNmax 1’ parameter to include reads that are uniquely mapped in the mouse mm10 genome and to exclude reads that are mapped to more than one locus. To control the mapping specificity and potential SNPs, we used the ‘—outFilterMismatchNmax 3’ parameter to manage that the maximum number of mismatches per read-pair is three. The ‘—outFilterScoreMinOverLread 0.25’ parameter means that the read alignment will be output only if its score is higher than or equal to 0.25 when normalized to the read length. The ‘—outFilterMatchNminOverLread 0.25’ parameter means that the read alignment will be output only if the number of matched bases is higher than or equal to 0.25 when normalized to the read length. The number of reads aligned to the exon regions of each gene was quantified by a Perl script, which was described in detail in our previous work (46) and is available at our GitHub site (Jerry-Zhao/KS2022).

### RNA-seq normalization and comparison

RNA-seq normalization and comparison were performed using edgeR (26). Briefly, the table of read counts was imported into R into a data.frame using the read.table command. The data.frame was then converted into a list-based data object by the DGEList command. Lowly expressed genes (average count-per-million < 0.5) were excluded from the downstream analysis. The library size was calculated by the colSums command, and the read counts were normalized to library size using the calcNormFactors command in edgeR to obtain the tag per million (TPM) values. The common dispersion and tagwise dispersions were estimated using the edgeR estimateDisp command. We next used the quasi-likelihood methods with empirical Bayes shrinkage in edgeR to fit the TPM data into a quasi-likelihood negative binomial generalized log-linear model using the glmQLFit command. Lastly, we carried out the quasi-likelihood F-test to compare gene expression using the glmQLFTest command. The cutoff for a significant difference in gene expression was FDR < 0.05. The Gene Ontology enrichment analyses were performed using the DAVID (27), and the genes expressed in Neuro 2a cells were used as the background genes.

### RNA-seq PCA

The DESeq2 (50) was used to perform the PCA. Briefly, the table of read counts was converted into a DESeqDataSet object using the DESeqDataSetFromMatrix command. Next, the DESeq command was used to estimate the library size factors, estimate the dispersion, fit a Negative Binomial GLM model and preform the Wald statistics. We then used the rlog function to transform the count data into the log2 scale, minimize differences between samples for rows with small counts and normalize to library size. Lastly, plotPCA was used on the transformed data to perform the PCA analysis to check for batch effects and the similarity among samples. The top 1000 highly variable genes were used for the PCA analysis using the parameter of ‘ntop = 1000’.

### Fragments per kilobase of transcript per million mapped read pairs (FPKM) calculation

FPKM value was calculated using the edgeR TPM value to normalize to the length of the exonic region of the gene.

### GSEA

The GSEA analyses were performed using the GSEA software (28). The TPM values from edgeR were used as the input files. The msigdb.v7.4.symbols.gmt was used as the gmt file. The Mouse_Gene_Symbol_Remapping_MSigDB.v7.0.chip was used as the chip file. The parameters of ‘1000 permutation’, ‘collapse’ and ‘permutation type: gene_set’ were selected.

### Motif enrichment analysis

The motif enrichment analysis was carried out using the ‘findMotifs.pl’ command in Homer (29). The input file contains the gene Ensembl IDs of the 1052 genes. The parameters used were ‘mouse -start -1000 -end 1000 -len 8,10,12 -p 30’. The *P*-value <1e-12 was used as the cutoff to identify enriched motifs from the Homer *de novo* Motif Results.

### ChIP-seq data and analysis

The FASTQ files of the ChIP-seq data were downloaded from the EMBL-EBI European Nucleotide Archive database. Bowtie (51) was used to align the FASTQ files to the mouse mm10 genome by the parameters of ‘-v 2 -m 1 -p 40’. The ‘samtools view’ (52) was used to convert the sam files into bam files. The bamCoverage (53) was used to convert the bam files into bw files by the parameters of ‘--binSize 10 -p 40’. Integrative genomics viewer (IGV) (54) was used to visualize the bw files. The 1000 control genes were selected from the genes expressed in Neuro 2a cells, and the ‘int rand’ function in Perl was used.

### Data plotting and statistical analysis

Most of the data plotting and statistical analysis were carried out using the R version 4.0.4. The ‘pheatmap’ in R was used to plot the heatmaps. The ‘t.test’ in R was used to carry out the *t*-test. The ‘cor’ in R was used to calculate the Pearson’s correlation coefficient.

### Availability

All computational scripts used in this study are available in the GitHub repository (https://github.com/Jerry-Zhao/KS2022).

### Accession numbers

The RNA-seq data of raw and processed files generated in this study have been deposited with the NCBI Gene Expression Omnibus under the accession number of GSE201401. The accession numbers for publicly available ChIP-seq data are GSM1693007 (Sox9), GSE91043 (H3K4me3 and H3K27ac), GSE69949 (Sox10) and GSE164360 (Chd7). The accession number for publicly available RNA-seq data is GSE164360 (Chd7 WT and knockout).

## Supplementary Material

Figure_Supple_revise_ddac172

Moon_Table_S1_ddac172

Moon_Table_S2_ddac172

Moon_Table_S3_ddac172
