# Supplementary material for: Convergent biological pathways underlying the Kallmann syndrome-linked genes Hs6st1 and Fgfr1
Source: Hum Mol Genet. 2022 Jul 28;31(24):4207–16. doi: 10.1093/hmg/ddac172 (PMC9759331; doi:10.1093/hmg/ddac172)
Supplement: Figure_Supple_revise_ddac172 [file figure_supple_revise_ddac172.pdf]

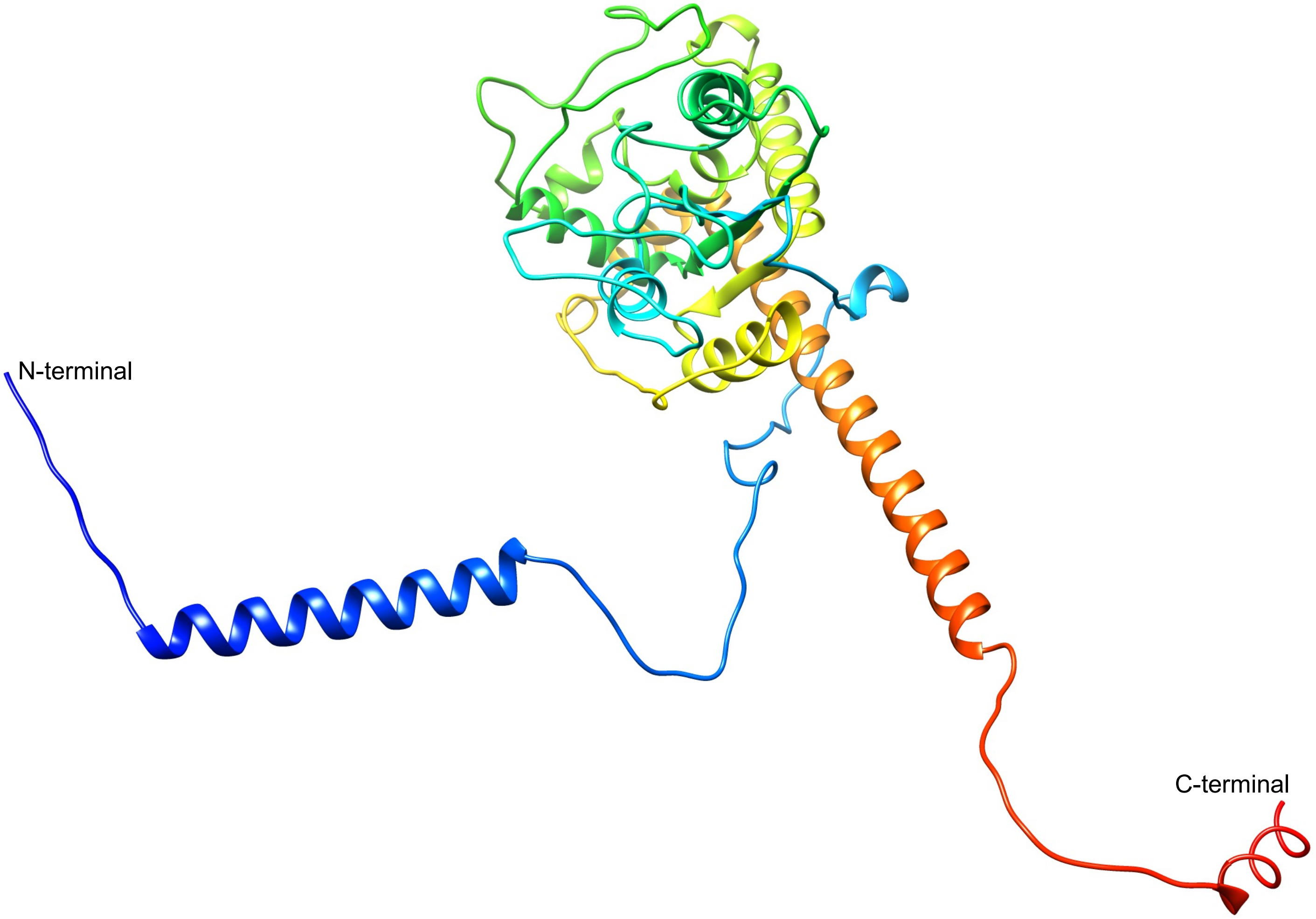


```
      α1                                                    α2
Human MRRRRAGGRTMVERASKFVLVVAGSVCFMLILYQYAGPGLSLGAPGGRAPPDDLDLFPTPDPHYEKKYYFPVREL 75
Mouse MRRRRAGGRTMVERASKFVLVVAGSACFMLILYQYAGPGLSLGAPGGRVPPDDLDLFPTPDPHYEKKYYFPVREL 75

      β1  α3
Human ERSLRFDMKGDDVIVFLHIQKTGGTTFGRHLVQNVRLEVPCDCRPGQKKCTCYRPNRRETWLFSRFSTGWSCGLH 150
Mouse ERSLRFDMKGDDVIVFLHIQKTGGTTFGRHLVQNVRLEVPCDCRPGQKKCTCYRPNRRETWLFSRFSTGWSCGLH 150

      α4  β2  α5
Human ADWTELTNCVPGVLDRRDSAALRTPRKFYYITLLRDPVSRYLSEWRHVQRGATWKTSLHMCDGRTPTPEELPPCY 225
Mouse ADWTELTNCVPGVLDRRDPAGLRSPRKFYYITLLRDPVSRYLSEWRHVQRGATWKTSLHMCDGRTPTPEELPPCY 225

      α6  α7  α8  β3  α9
Human EGTDWSGCTLQEFMDCPYNLANNRQVRMLADLSLVGCYNLSFIPEGKRAQLLLESAKKNLRGMAFFGLTEFQRKT 300
Mouse EGTDWSGCTLQEFMDCPYNLANNRQVRMLADLSLVGCYNLSFIPESKRAQLLLESAKKNLRGMAFFGLTEFQRKT 300

      α10  α11
Human QYLFERTFNLKFIRPFMQYNSTRAGGVEVDEDTIRRIEELNDLDMQLYDAKDLFQQRYQYKRQLERREQRLRSR 375
Mouse QYLFERTFNLKFIRPFMQYNSTRAGGVEVDEDTIRHIEELNDLDMQLYDAKDLFQQRYQYKRQLERREQRLRNR 375

Human EERLLHRAKEALPREDADEPGRVPTEDYMSHIIEKW 411
Mouse EERLLHRSKEALPREDPEEPGRVPTEDYMSHIIEKW 411
```

Supplemental Figure S1. Protein structure of the human HS6ST1 predicted by the AlphaFold. α, α-helix. β, β-strand.

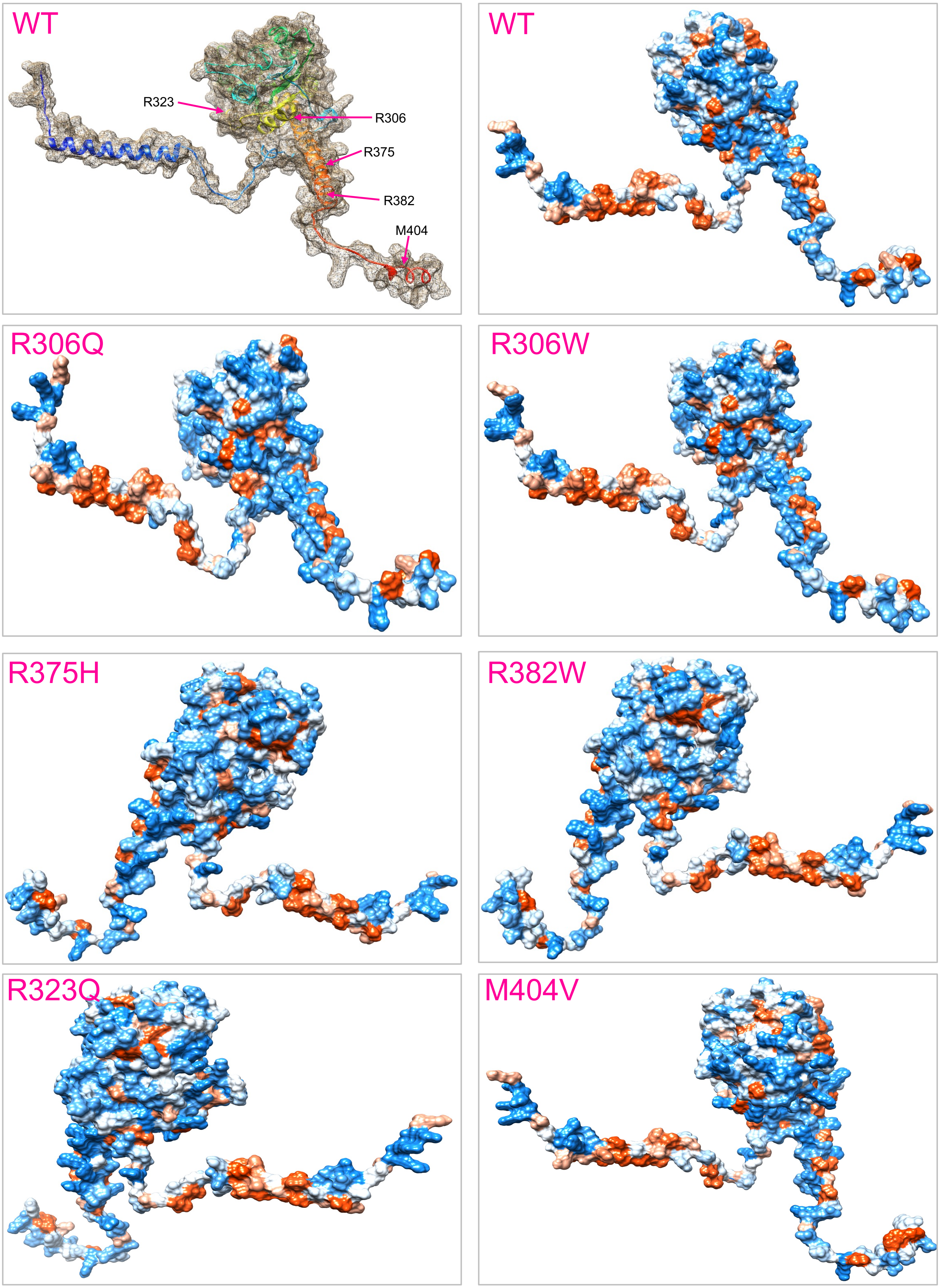

Supplemental Figure S2. Protein structures of the WT and mutant human HS6ST1 predicted by the AlphaFold.

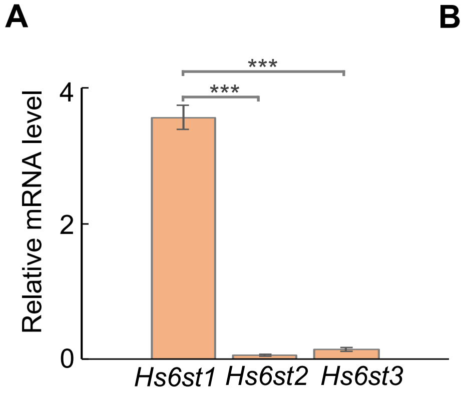

B

	Total read pairs	Uniquely mapped read pairs
Ctrl replicate 1	88,036,346	76,381,000 (86.76%)
Ctrl replicate 2	54,384,194	46,598,448 (85.68%)
Ctrl replicate 3	40,630,458	34,720,575 (85.45%)
siHs6st1 replicate 1	22,058,825	19,032,190 (86.28%)
siHs6st1 replicate 2	30,229,727	26,121,458 (86.41%)
siHs6st1 replicate 3	23,244,310	20,113,225 (86.53%)
siFgfr1 replicate 1	26,938,712	23,214,144 (86.17%)
siFgfr1 replicate 2	20,623,653	18,224,942 (88.37%)
siFgfr1 replicate 3	39,069,745	33,166,410 (84.89%)
siFgfr1 replicate 4	156,060,365	135,088,193 (86.56%)

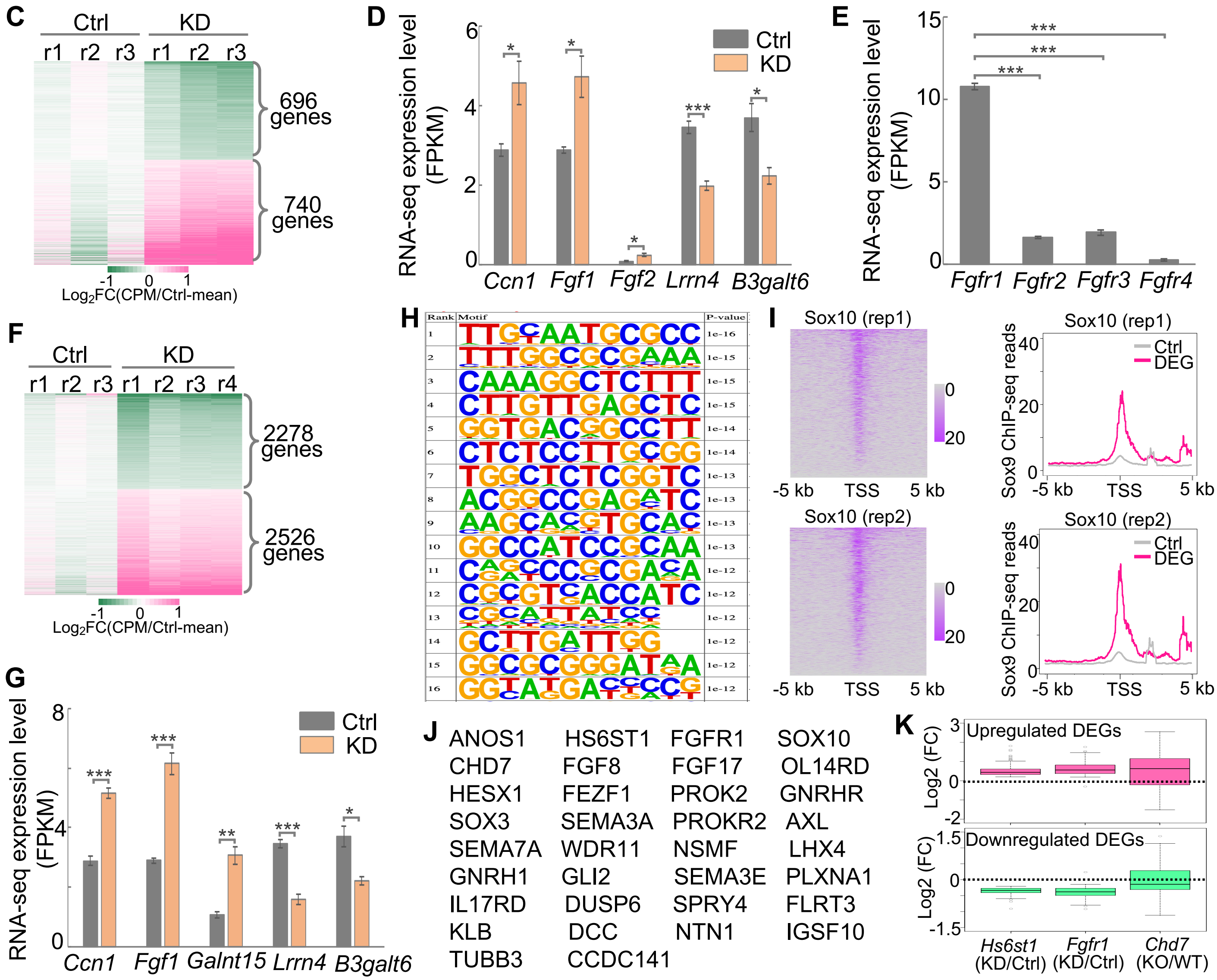

Supplemental Figure S3. siRNA knockdown (KD) of Hs6st1 (siHs6st1) and Fgfr1 (siFgfr1) in Neuro 2a cells followed by RNA sequencing (RNA-seq). **A**, qPCR results of the expression levels of the three *Hs6st* genes in Neuro 2a cells. **B**, Statistics of the sequencing and mapping of the RNA-seq data. **C**, Heatmap of the fold changes of the differentially expressed genes upon *Hs6st1* knockdown (KD). r1, biological replicate 1. **D**, RNA-seq expression levels of five genes in the control (Ctrl) and Hs6st1 knockdown (KD). **E**, RNA-seq expression levels of the four *Fgfr* genes in Neuro 2a cells. **F**, Heatmap of the fold changes of the differentially expressed genes upon *Fgfr1* knockdown (KD). **G**, RNA-seq expression levels of five genes in the control (Ctrl) and *Fgfr1* knockdown (KD). **H**, The identified *de novo* motifs by Homer. **I**, Heatmaps and line plots showing the Sox10 ChIP-seq signals at the promoter regions. **J**, The thirty-four Kallmann syndrome-linked genes. *, *P-value* < 0.05; **, *P-value* < 0.01; ***, *P-value* < 0.001; one-tailed t-test. **K**, The expression changes (log2FC) of overlapping DEGs in *Hs6st1* KD, *Fgfr1* KD, and *Chd7* knockout (KO).
